# Knowledge, attitudes and behaviours of nurses about antibiotic use and antibiotic resistance in Oman

**DOI:** 10.1371/journal.pone.0342371

**Published:** 2026-05-18

**Authors:** Zainab Said Al-Hashimy, Barbara R. Conway, Sayer Al-Azzam, Reema Karasneh, Halima Ali Rashid Al Kiyumi, Stuart E. Bond, Kelly Atack, Raneem Saed Nofal, Mamoon A. Aldeyab

**Affiliations:** 1 Department of Clinical Pharmacy, Directorate of Pharmaceutical Care and Medical Stores, Khoula Hospital, Muscat, Oman; 2 Department of Pharmacy, School of Applied Sciences, University of Huddersfield, Huddersfield, United Kingdom; 3 Institute of Skin Integrity and Infection Prevention, University of Huddersfield, Huddersfield, United Kingdom; 4 Department of Clinical Pharmacy, Jordan University of Science and Technology, Irbid, Jordan; 5 Department of Basic Pathological Sciences, Faculty of Medicine, Yarmouk University, Irbid, Jordan; 6 Department of Medical Equipment, Directorate of Medical Materials Warehouses, Directorate General of Medical Supply, Muscat, Oman; 7 Pharmacy Department, Mid Yorkshire Teaching NHS Trust, Wakefield, United Kingdom; 8 Pharmacy Department, Leeds Teaching Hospitals NHS Trust, Leeds, United Kingdom; 9 Reading School of Pharmacy, University of Reading, Reading, United Kingdom; IMU: International Medical University, MALAYSIA

## Abstract

**Background:**

Antimicrobial resistance (AMR) is a significant global health challenge, with organizations worldwide emphasizing the importance of addressing inappropriate antibiotic use and resistance. The aim of this study was to investigate nurses’ knowledge, attitudes, and behaviour on antibiotic use and resistance in Oman.

**Methods:**

This cross-sectional study used a questionnaire designed by the European Centre for Disease Prevention and Control, which was distributed to nurses working in Oman’s Ministry of Health.

**Results:**

A total of 424 nurses responded to the survey. A total of 64.2% and 48.8% of the respondents accurately acknowledged the lack of efficacy of antibiotics against viruses and the common cold, respectively. Nevertheless, a significant majority of 93% of participants were able to provide accurate responses regarding excessive use of antibiotics and associated adverse effects. Out of the surveyed nurses, 59% demonstrated awareness of the Oman National Action Plan on antibiotic resistance. Of those who responded, 54.7% wanted to know which antibiotics are used for specific medical conditions, 52.1% wanted to know more about antibiotic resistance, 42% wanted to learn about the proper usage of antibiotics, and 30% were interested in the links between humans, animals, and environmental health.

**Conclusion:**

The study’s results should be used to enhance education and increase nurses’ capabilities and understanding regarding antibiotic use and antibiotic resistance. Strengthening capabilities, opportunities, and motivation is essential to empower nurses as frontline contributors in the global fight against antibiotic resistance.

## Introduction

Antimicrobial resistance (AMR) is a major health challenge affecting countries worldwide, a concern that several organizations have highlighted, focusing on the use and development of resistance to antibiotics [1]. The World Health Organization (WHO) ranks it as the top ten global health threats. This growing issue is a primary contributor to morbidity and mortality, with projections estimating it could lead to approximately 10 million deaths annually by the year 2050 [1–3]. The implementation of infection prevention and control programs (IPC) can reduce AMR. Therefore, enhancing IPC practice in Low and Middle Income (LMIC) healthcare settings could help prevent deaths associated with AMR annually [4].

Antimicrobial stewardship programs (AMS) are essential across all healthcare institutions. AMS can promote the appropriate use of antibiotics to reduce antibiotic resistance [5]. These programs involve multidisciplinary teams led by a physician, a microbiologist, a pharmacist, and nurses. The team is responsible for optimizing the use of antibiotics in the hospital, ensuring that all healthcare professionals adhere to the antimicrobial guidelines, and providing training and education on the necessary knowledge and tools [6,7].

Nurses play a vital role in antimicrobial stewardship programs, serving as essential communicators among physicians, pharmacists, laboratories, and patients [8]. Their direct involvement in patient care and antibiotic administration positions them as critical advocates for responsible antibiotic use [9]. Nurses employ various strategies in antimicrobial stewardship, including screening, microbiological sampling, administration, monitoring, discharge, surveillance, infection prevention and control, and collaboration with multidisciplinary teams [9,10].

A significant knowledge gap exists among nurses regarding the use of antibiotics and antibiotic resistance, which should be addressed [11]. To address this gap, education and training, as well as acknowledgment of nurses’ contributions, are necessary to optimize their roles in AMS [12]. In Oman, there was a lack of knowledge about nosocomial infections among student nurses [13]. Little literature has specifically evaluated nurses’ knowledge, attitudes, and behaviours regarding antibiotic use and resistance. In Oman, nurses contribute at all levels of the health system, from primary health centers to tertiary hospitals, with responsibilities ranging from preventive and community-based care to specialized clinical practice, leadership, and research. Their roles are supported by a structured educational pathway that progresses from diploma to bachelor’s and postgraduate specialization, enabling nurses to serve in diverse settings and assume responsibilities that match the complexity of care and the needs of the population.

Therefore, this study aimed to assess nurses’ knowledge gaps to guide targeted interventions. The COM-B model (Capability, Opportunity, Motivation – Behaviour) was used. Capability refers to nurses’ knowledge and skills [11]. Opportunities include external factors such as guidelines and training [14]. Motivation is influenced by professional responsibility and habitual factors [15]. Addressing these components can inform practical interventions to promote responsible antibiotic use.

## Methodology

### Study design

This was a cross-sectional survey study conducted from 18 January to 14 June 2023 involving nurses registered with Oman’s Ministry of Health (MOH). The survey used an adapted European Centre for Disease Prevention and Control (ECDC) questionnaire [16,17], Which has been used in previous studies, and permission to use the questionnaire was obtained from ECDC. Minor contextual adjustments were made to ensure relevance to the healthcare system in Oman while maintaining the original structure and intent of the questions. The original instrument was developed in English. The study survey was available in both English and Arabic to accommodate all participating nurses. The Arabic version was developed using a forward translation approach, in which the English questions were translated into Arabic by bilingual clinical experts. To ensure the meaning remained the same in both languages, a panel of bilingual specialists reviewed the final version for clarity and accuracy. During the study, participants used a Qualtrics platform to choose their preferred language before answering. The original ECDC instrument has demonstrated content validity in prior applications. As the questionnaire was adopted without structural modification and consisted primarily of knowledge-based items, internal consistency reliability testing was not re-assessed in the present study. The survey was distributed via the web-based Qualtrics software through the MOH’s email system and WhatsApp groups. The invitation message included information about the study objectives, voluntary participation, and confidentiality of responses. Participants were informed that completing the questionnaire implied their consent to participate in the study. Measures were implemented to ensure that each participant provided only one response. Participation was voluntary, and responses were anonymized and kept confidential.

The research approved by the Health Studies and Research Approval Committee in Muscat, Oman (Moh/DGPS/CSR/PRO/approved/137/2022) and the University of Huddersfield Research Integrity and Ethics Committee (SAS-SRIEC-21.12.22–1).

### Survey tool

The behaviour is affected by an individual’s competence, opportunity, and motivation; therefore, the questionnaire was developed based on the COM-B behavioural change theory. The survey had seven sections. Section one collected demographic data (age, gender, governorate, profession, workplace, and experience). Section two measured respondents’ knowledge using eight true-or-false questions, including awareness of antibiotic usage, inappropriate usage, adverse effects, antibiotic resistance, and the application of antibiotics in the agricultural sector. Correct responses received one point on this scale, which ranges from 0 to 8. The third section used a 5-point Likert scale, along with “not applicable” or “I don’t understand” options, covering resistance in food and the environment, perceived knowledge, opportunities for prudent antibiotic use and infection management, and opportunities to support AMR control.

Section four asked three questions on the frequency of opportunities to provide antibiotics or resources, with response options ranging from more than once a day, more than once a week, once a day, once a week, never, rarely, “not applicable”, and “I do not remember”.

The fifth section covered resources used in managing infections. The sixth section enquired if nurses received information on avoiding unnecessary antibiotic administration and whether it changed their views and practices. The seventh section explored sources of information for improving antibiotic administration, and awareness of national initiatives and action plans within the country, as well as topics of interest to nurses.

### Statistical analysis

A sample size of 375 was calculated using the Raosoft calculator (http://www.raosoft.com/samplesize.html), based on a 5% margin of error, a 95% confidence level (Z = 1.96), and a 50% response distribution. The calculation was based on a total of 14,361 nurses registered with the Ministry of Health in Oman, as reported in the MOH annual health report 2021. The calculated sample size was considered sufficient to provide a representative estimate of nurses working within the MOH healthcare system. Data were analyzed using IBM SPSS Statistics version 28.0 (IBM Corp., Armonk, NY, USA). In this study, only fully completed survey responses were included in the final analysis (n = 424). To ensure data quality, only surveys that were fully completed and saved by the platform were included in the final analysis. Incomplete entries, where participants exited before finishing, were automatically excluded during the data cleaning phase. Consequently, there were no missing values in the final dataset (n = 424) used for statistical analysis. Sociodemographic variables were reported as frequencies and percentages. Knowledge scores were summarized using the median and interquartile range and analyzed using the Mann-Whitney U test and the Kruskal-Wallis rank-sum test. In contrast, differences in full knowledge scores were assessed using chi-square or Fisher’s exact test, both two-tailed. The p-value threshold for statistical significance has also been specified. Univariable and multivariable linear regression analyses were performed to identify independent predictors of participants’ knowledge scores. Initially, all demographic variables were screened using univariable analysis. Variables with p-values < 0.20 or identified as clinically relevant were included in the final multivariable model to adjust for potential confounding. Statistical significance was defined as a two-tailed p-value < 0.05, and results were reported as unstandardized coefficients (B) with 95% confidence intervals (CI). To ensure the validity of the regression model, statistical assumptions, including normality, homoscedasticity, and multicollinearity (via Variance Inflation Factor, VIF), were verified and met.

## Results

### Key findings

#### Demographics data.

A 424 nurses responded to the survey, 46.7% were originating from Muscat Governorate, and 49.3% of participants were aged 24–35 years. Most respondents (63.7%) were general nurses, and 81.1% worked in hospitals. There was a 31.8% participant-to-experience ratio between 11 and 15 years (Table 1).

**Table 1:** Nurses’ sociodemographic characteristics (n = 424).

Variable	n (%)
**Gender**	
Female	369 (87.0)
Male	55 (13.0)
**Age**	
24-35 years	209 (49.3)
36-45 years	186 (43.9)
46-55 years	24 (5.7)
56-65 years	5 (1.2)
**Governorate**	
Ad Dakhiliyah	55 (13.0)
Ad Dhahirah	9 (2.1)
Al Batinah North	12 (2.8)
Al Batinah South	21 (5.0)
Al Buraymi	16 (3.8)
Al Wusta	59 (13.9)
Ash Sharqiyah North	6 (1.4)
Ash Sharqiyah South	32 (7.5)
Dhofar	4 (0.9)
Musandam	12 (2.9)
Muscat	198 (46.7)
**Profession**	
BSc. Nurse	102 (24.1)
BSc. Nurse with Specialism	52 (12.3)
General Nurse (Diploma)	270 (63.7)
**Place of practice**	
Hospital (any hospital type)	344 (81.1)
Primary health center	66 (15.6)
Secondary care center (polyclinic or dialysis center)	14 (3.3)
**Years of practice**	
0-2 years	26 (6.1)
3-5 years	47 (11.1)
6-10 years	104 (24.5)
11-15 years	135 (31.8)
16-20 years	85 (20.0)
21-25 years	21 (5.0)
>25 years	6 (1.4)

### Actual capability

Table 2 displays the nurses’ actual knowledge across eight questions: 64.2% and 48.8% correctly identified the ineffectiveness of antibiotics against viruses and colds, 93.2% recognized risks of overuse, and 74.1% acknowledged increased resistance. 58.5% understood the potential for spread of resistance, 66.3% knew that healthy individuals can carry resistant organisms, and only 21% were aware that antibiotic use in agriculture is legally restricted, indicating moderate overall awareness of AMR.

**Table 2:** Respondents’ actual knowledge (n = 424).

Key Knowledge Question	Correct Answer	True* n (%)	False* n (%)	Unsure* n (%)
Antibiotics are effective against viruses.	False	130 (30.7)	272 (64.2)	22 (5.2)
Antibiotics are effective against the common cold.	False	171 (40.3)	207 (48.8)	46 (10.8)
The unnecessary use of antibiotics makes them become ineffective.	True	397 (93.6)	18 (4.2)	9 (2.1)
Taking antibiotics has associated side effects or risks such as diarrhea, colitis, allergies.	True	395 (93.2)	10 (2.4)	19 (4.5)
Every person treated with antibiotics is at an increased risk of antibiotic-resistant infection.	True	314 (74.1)	66 (15.6)	44 (10.4)
Antibiotic-resistant bacteria can spread from person to person.	True	248 (58.5)	123 (29.0)	53 (12.5)
Healthy people can carry antibiotic-resistant bacteria.	True	281 (66.3)	74 (17.5)	69 (16.3)
The use of antibiotics to stimulate growth in farm animals is legal in Oman.	False	58 (13.7)	89 (21.0)	277 (65.3)

*Represents the number of respondents (percentage) who answered as true, false, or unsure.

Table 3 presents the multivariable linear regression model, while the univariable linear regression results are provided in Supplementary S1 Table in S3 File. The final model demonstrated a statistically significant (*F* = 4.546, *p* < 0.001), with an Adjusted *R*^*2*^ of 0.091. This indicates that the included demographic predictors collectively account for 9.1% of the variance in the participants’ knowledge scores. After adjusting for potential confounders, gender and years of practice emerged as the only independent predictors of knowledge. Specifically, male nurses achieved significantly higher knowledge scores than their female counterparts (*B* = 0.678, 95% CI: 0.271–1.084, *p* = 0.001). Regarding professional experience, staff with 16–20 years of practice demonstrated positive association with knowledge (*B* = 1.116, 95% CI: 0.419–1.813, *p* = 0.002), followed by those with 21–25 years (B = 1.053, *p* = 0.021) and >25 years (B = 1.683, *p* = 0.023) of experience. Notably, while age groups and professional specialisms were significant in univariable screening, they lost statistical significance in the multivariable model (*p* > 0.05), suggesting that the observed effects of age were largely confounded by accumulated years of professional practice. Collinearity diagnostics confirmed the model’s stability, with all Variance Inflation Factors (VIFs) remaining below 5.0 model’s stability, with all Variance Inflation Factors (VIFs).

**Table 3:** Multivariable linear regression analysis of the relationship between knowledge scores and demographic characteristics of respondents.

		Unstandardized Coefficients		Standardized Coefficients	t	Sig.		95.0% Confidence Interval for B
		B	Std. Error	Beta			Lower Bound	Upper Bound
**Gender**	Female							
	Male	0.678	0.207	0.155	3.279	**0.001**	0.271	1.084
**Age**	24-35 years							
	36-45 years	0.325	0.184	0.110	1.765	0.078	−0.037	0.688
	46-55 years	0.511	0.357	0.080	1.432	0.153	−0.191	1.213
	56-65 years	−0.906	0.707	−0.066	−1.281	0.201	−2.295	0.484
**Profession**	BSc. Nurse							
	BSc. Nurse with Specialism	0.160	0.252	0.036	0.636	0.525	−0.335	0.656
	General Nurse (Diploma)	−0.283	0.172	−0.093	−1.646	0.101	−0.621	0.055
**Years of practice**	0-2 years							
	3-5 years	0.573	0.343	0.122	1.668	0.096	−0.102	1.247
	6-10 years	0.590	0.310	0.173	1.902	0.058	−0.020	1.200
	11-15 years	0.538	0.311	0.170	1.727	0.085	−0.074	1.150
	16-20 years	1.116	0.355	0.304	3.147	**0.002**	0.419	1.813
	21-25 years	1.053	0.456	0.155	2.308	**0.021**	0.156	1.949
	>25 years	1.683	0.737	0.135	2.282	**0.023**	0.234	3.132

B = unstandardized coefficient; SE = standard error; beta = standardized coefficient; t = t-statistic; p = p-value; CI = confidence interval.

S2 Table presents the differences in median knowledge scores across demographic groups. The bivariate analysis revealed that gender was a significant factor, with male nurses achieving higher median scores than female nurses (6.0 vs 5.0; *p* = 0.008). Similarly, age played a critical role (*p* < 0.001), with the 46–55-year-old age group demonstrating the highest knowledge levels. In Oman, nurses generally complete a diploma followed by a bachelor’s degree, with some pursuing further specialization. Nurses with specialisms—those with higher diplomas or additional training in areas such as emergency care, critical care, infection control, maternity, or management—showed significant median knowledge scores (p = 0.008; S2 Table in S3 File). Finally, clinical experience is significantly related to expertise, with nurses with over 25 years of practice recording the highest median score (6.5; *p* < 0.001).

S3 Table in S3 File compares demographic factors between participants with full knowledge (n = 15) and those with incomplete scores (n = 409). Statistical analysis using Fisher’s Exact test revealed that gender (*p* = 0.007), age (*p* = 0.024), and governorate (*p* = 0.036) were significantly associated with achieving a perfect score. Notably, while males represented a small portion of the total sample, they accounted for 40% of the full-knowledge group. In contrast, profession, place of practice, and years of experience did not significantly influence the likelihood of achieving a perfect score (p > 0.05).

### Perceived capability and opportunity

Data in Table 4 show that 79.5% of nurses agree or strongly agree that they are aware of antibiotic resistance, 81.8% indicate they have sufficient information regarding antibiotic use, and 86.4% report being able to educate others about antibiotic resistance.

**Table 4:** Nurses’ perceived knowledge (n = 424).

	SA n (%)	A n (%)	D n (%)	SD n (%)	N/A n (%)	U n (%)	IDU n (%)
Perceived Knowledge							
I know what antibiotic resistance is	108 (25.5)	229 (54.0)	10 (2.4)	25 (5.9)	6 (1.4)	26 (6.1)	20 (4.7)
I know what information to give to individuals about prudent use of antibiotics and antibiotic resistance	79 (18.6)	268 (63.2)	19 (4.5)	13 (3.1)	5 (1.2)	36 (8.5)	4 (0.9)
I have sufficient knowledge about how to use antibiotics appropriately for my current practice	91 (21.5)	275 (64.9)	13 (3.1)	14 (3.3)	2 (0.5)	29 (6.8)	0 (0)
Opportunity							
I have easy access to the guidelines I need on managing infections	67 (15.8)	241 (56.8)	41 (9.7)	11 (2.6)	12 (2.8)	47 (11.1)	5 (1.2)
I have easy access to the materials I need to give advice on prudent antibiotic use and antibiotic resistance	49 (11.6)	230 (54.2)	60 (14.2)	12 (2.8)	10 (2.4)	54 (12.7)	9 (2.1)
I have good opportunities to provide advice on prudent antibiotic use to individuals	48 (11.3)	264 (62.3)	41 (9.7)	15 (3.5)	14 (3.3)	40 (9.4)	2 (0.5)
Motivation to initiate antibiotic prescriptions							
I know there is a connection between my administering of antibiotics and emergence and spread of antibiotic-resistant bacteria	69 (16.3)	239 (56.4)	43 (10.1)	19 (4.5)	0 (0)	41 (9.7)	13 (3.1)
I have a key role in helping control antibiotic resistance	75 (17.7)	231 (54.5)	26 (6.1)	14 (3.3)	15 (3.5)	57 (13.4)	6 (1.4)
One Health: environmental and animal health factors that are important in contributing to antibiotic resistance in bacteria from humans							
Environmental factors such as wastewater in the environment are important in contributing to antibiotic resistance in bacteria from humans	34 (8.0)	193 (45.5)	46 (10.8)	13 (3.1)	22 (5.2)	84 (19.8)	32 (7.5)
Excessive use of antibiotics in livestock and food production is important in contributing to antibiotic resistance in bacteria from humans	44 (10.4)	186 (43.9)	38 (9.0)	20 (4.7)	15 (3.5)	86 (20.3)	35 (8.3)

Abbreviations: SA: strongly agree; A: agree; D: disagree; SD: strongly disagree; N/A: not applicable; U: undecided; IDU: I do not understand.

Over 72.6% and 65.8% of the respondents strongly agreed that they had easy access to guidelines and materials to advise others regarding resistance. In comparison, 73.6% reported opportunities to educate individuals about prudent antibiotic use (Table 4).

### Motivation and one health

Seventy-two percent of respondents agreed that they are aware of the link between antibiotic use and the spread of resistance, and 72.2% acknowledged their key role in controlling it (Table 4).

Table 4 presents a One Health-related question, revealing that around 53% of respondents agreed that excessive antibiotic use in livestock and food production, as well as environmental factors, contributes to antibiotic resistance in bacteria affecting humans, reflecting the One Health concept, which emphasizes the interconnection between people, animals, plants, and their shared environment. Meanwhile, 65.3% were unsure about the legality of antibiotic use on animal farms (Table 2).

### Behaviour

Table 5 presents nurses’ behaviour in promoting the appropriate use of antibiotics: 42.2% administered antibiotics at least once daily, 65.8% did not provide adequate information on proper use and infection management, and 39.3% offered little to no guidance on responsible use.

**Table 5:** The frequency with which nurses provided antibiotics or resources related to the prudent use of antibiotics (n = 424).

Item	>QD n (%)	>QW n (%)	NVR n (%)	R n (%)	QD n (%)	QW n (%)	N/A n (%)	IDR n (%)
How often did you administer antibiotics during the last one week?	123 (29.0)	40 (9.4)	91 (21.5)	48 (11.3)	56 (13.2)	24 (5.7)	25 (5.9)	17 (4.0)
How often did you give out resources (e.g., leaflets or pamphlets) on prudent antibiotic use or management of infections to individuals during the last one week?	26 (6.1)	13 (3.1)	150 (35.4)	129 (30.4)	25 (5.9)	28 (6.6)	21 (5.0)	32 (7.5)
How often did you give out advice related to prudent antibiotic use or management of infections to an individual during the last one week?	62 (14.6)	41 (9.7)	84 (19.7)	83 (19.6)	35 (8.3)	72 (17.0)	17 (4.0)	30 (7.1)

Abbreviations: QD: once a day; QW: once a week; NVR: never; R: rarely; N/A: not applicable; IDR: I do not remember.

Table 6 shows that 55% of the nurses received information on avoiding unnecessary antibiotic use, with 92.7% reporting it changed their opinions and 91.7% stating it influenced their antibiotic administration practices.

**Table 6:** Number of respondents (%) who received information on avoiding unnecessary administration of antibiotics and, of those, the number (%) reporting that the information contributed to changing their views or practice.

Questions	Yes	No	Unsure
	*n* (%)	*n* (%)	*n* (%)
In the last 12 months, do you remember receiving any information about avoiding unnecessary administration of antibiotics? (n = 424)	233 (55.0)	127 (30.0)	64 (15.1)
Did the information change your views about avoiding unnecessary administration of antibiotics? (n = 233)	216 (92.7)	8 (3.4)	9 (3.9)
Based on the information you received, have you changed your practice on the administration of antibiotics? (n = 216)	198 (91.7)	5 (2.3)	13 (6.0)

**Fig 1:**
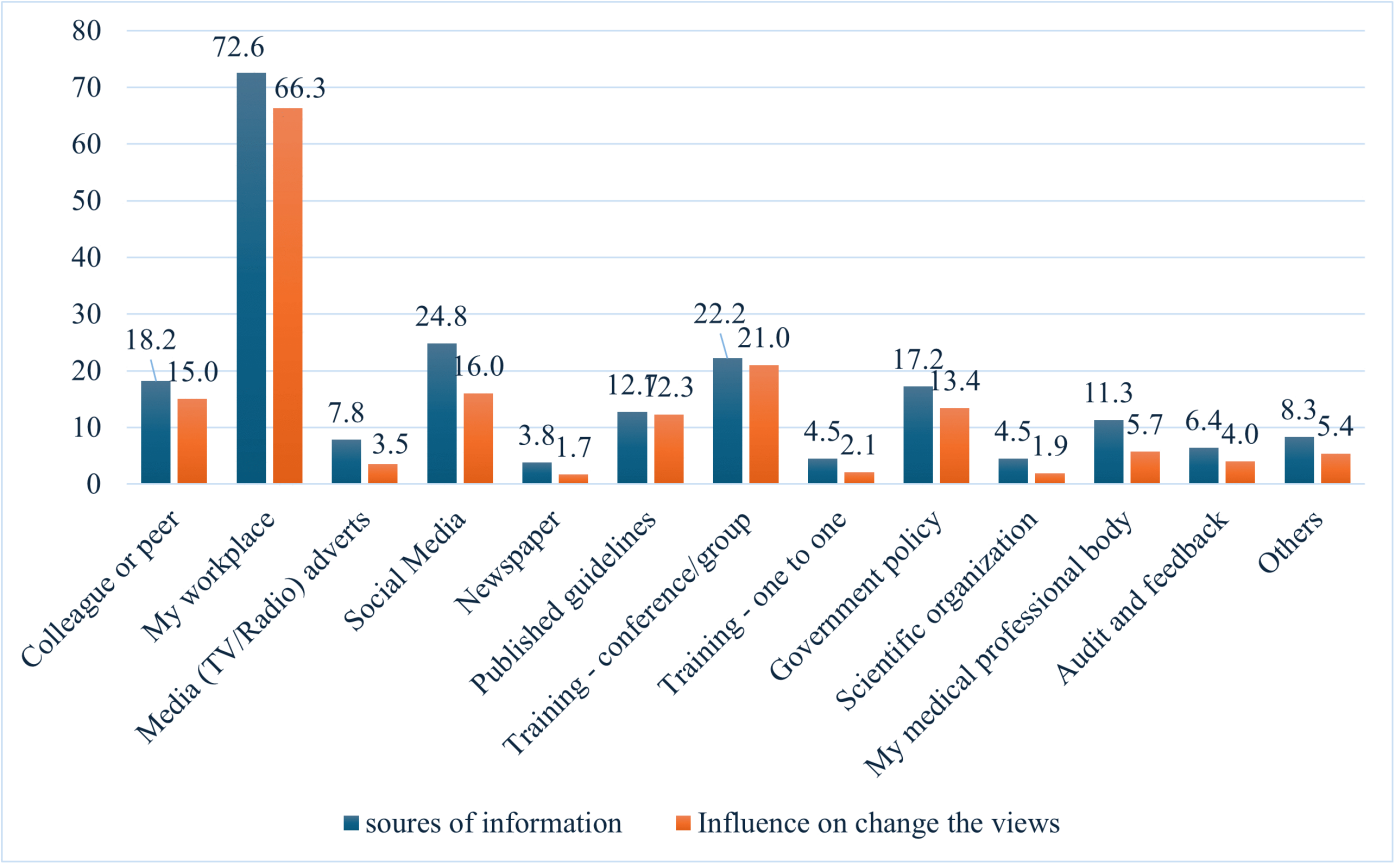
Sources of information about avoiding unnecessary administration of antibiotics and their influence on changing nurses’ views (n = 424).

The survey reveals that over 72.6% of participants received workplace advice on minimizing the use of unnecessary antibiotics, leading to a change in the views of 66.3%. Over 24.8% gained knowledge via social media, with only 16% altering their practices (Fig 1).

### Awareness of the Oman National Action Plan and National Initiatives

Among the nurses surveyed, 59% showed awareness of the Oman National Action Plan on antibiotic resistance, 5% were unaware of it, and 36% were unsure whether the country had a National Action Plan.

Surveys on antibiotics and resistance initiatives revealed that professional organizations educated 43.3% of respondents, 38.7% were aware of national or regional infection management protocols, and 32.3% were familiar with healthcare toolkits. Approximately 23.1% of respondents were aware of World Antibiotic Awareness Week, and 10.6% indicated no awareness of these activities (S4 Table in S3 File).

### Resources for managing infections and information gaps highlighted by nurses

S5 Table in S3 File shows that nurses primarily used clinical practice guidelines (58.7%) to manage infection, followed by continuing education (36.6%), consultation with infection specialists (34.9%), social media (22.6%), and 2.8% reported using no resources.

S1 Fig indicates nurses’ information needs: 54.7% wanted guidance on antibiotics for specific medical conditions, 52.1% wanted to know about antibiotic resistance, 41.5% wanted to understand how to use antibiotics, 30.4% requested to learn how to get prescriptions, and 30% needed to know how the link between human, animal, and environmental health.

The results of this study highlighted the need to enhance understanding of AMR and to increase nurses’ knowledge.

## Discussion

### Knowledge and awareness of antimicrobial resistance among nurses

The study was carried out with nursing professionals working across healthcare institutions within the Ministry of Health in Oman. Only 15 of 424 (3.5%) nurses completed all actual knowledge questions correctly in our survey. 45.1% of nurses reported not receiving or recalling instructions on antibiotic avoidance in the past year. Clinical practice guidelines are used by 58.7% of nurses, and 72.6% find them easy to access.

Our findings show that nurses demonstrated a moderate level of knowledge about antibiotic resistance. In contrast, a European study revealed a high level of knowledge, but an opposite result regarding the amount of sufficient information they have about antibiotic use in their practice [17]. A study conducted in Saudi Arabia revealed moderate knowledge and positive attitudes, but no significant relationship between them [11]. Higher education and training may support more positive attitudes, but not better knowledge [11]. In our study, 51.1% of nurses incorrectly believed that antibiotics are effective against the common cold. This result is highly consistent with a similar study in Saudi Arabia, which found that 53% of participants held the same misconception [18]. These findings suggest that a significant number of healthcare providers in the region still struggle to distinguish between viral and bacterial infections.

Regarding the use of antibiotics for viral infections, 64% of nurses in our study correctly identified them as ineffective. This is a better result than a similar study in India, where 54% of participants incorrectly agreed that antibiotics should be used for viral infections [19]. While our findings show a higher level of awareness, there remains a 36% knowledge gap that needs to be addressed through education.

Our findings indicate that above 53% of nurses believe that environmental factors and the overuse of antibiotics in food production significantly contribute to antibiotic resistance in humans.

At the same time, the percentage is higher, at around 88%, regarding the excessive use in livestock and around 67% agreed that environmental factors contribute to AMR [20]. Our nurses have a limited understanding of the prohibited use of antibiotics in animal growth, compared to 47% in a similar Italian study [20].

The study revealed that 41% of nurses had limited awareness of the Oman National Action Plan against antibiotic resistance, underscoring the need for all nurses to be informed about the plan and the use of antibiotics in agriculture [21]. A study conducted in Italy among ICU staff also revealed low awareness of national and international AMR campaigns [20]. Similarly, our findings suggest that the low awareness of AMR initiatives both nationally and internationally needs to be addressed.

### Nurses’ role and barriers in antimicrobial stewardship

Our findings suggest that nurses play a crucial role in mitigating antibiotic resistance. Previous studies have highlighted their role in antibiotic use and the implementation of antimicrobial stewardship programs [22,23]. Evidence from the literature shows that nurses demonstrate strengths in assessing adverse drug reactions, obtaining cultures, and educating patients; however, they often report low confidence in interpreting microbiology results [24]. To enhance their contributions to AMS, clear role definitions, leadership support, and additional research are needed [25,26]. In our survey, nurses reported gaps in knowledge and confidence related to antibiotics. However, the literature also identifies broader barriers to nurses’ involvement in AMS, including patient influence, environmental factors, underlying beliefs, attitudes, and lack of cooperation from prescribers [27,28].  These findings emphasize the importance of educational and training programs in strengthening nurses’ clinical assessment and communication skills with both patients and healthcare teams, which may improve patient outcomes and enhance nurses’ contributions to the care of patients with AMS [27,28].

In this study, 29% of nurses reported administering antibiotics more than once daily. While they support optimal use, their integration and competency in AMS remain limited, highlighting the need for stronger education, training, and institutional support [29].

### Education, training, and future directions

In Oman, formal training specifically dedicated to antimicrobial resistance (AMR) is not currently a standardized part of the national nursing curriculum. Instead, education is primarily provided through hospital-based Infection Prevention and Control (IPC) programs and Antimicrobial Stewardship (AMS) activities. The availability of these resources varies by workplace; nurses in large tertiary hospitals often have more access to specialized workshops and bedside clinical teaching compared to those working in primary healthcare centers.

Our findings highlight the importance of nurses enhancing their understanding of antibiotic use and resistance. Therefore, specialized training is needed to equip them with the knowledge, skills, and attitudes required to combat AMR, with WHO and CDC programs offering valuable support in areas such as antimicrobial stewardship, infection prevention and control, surveillance, and communication skills [26,30,31].

Research has shown that nurses’ training in AMS activities leads to improvements in AMS behaviours [32]. As our data indicate that nurses require further education and support to optimize antibiotic use, enhancing training in areas such as microbiology and pharmacy, alongside leadership support, may encourage stronger AMS involvement [33]. The nurses felt excluded from responsibilities and training related to AMR [34]. Although they are aware of the importance of hand hygiene, they hold a negative perception of it and struggle to maintain good hand hygiene standards in practice [35].

Our results showed that over 72.6% of nurses received information about the unnecessary administration of antibiotics in their workplace, and around 66.3% changed their view accordingly. Nursing facilities lack formal AMS and access to infectious disease experts, with programs often led by nurses or infection preventionists, and limited involvement from pharmacists and medical directors, leading to high antibiotic use and complex decision-making [36]. Communication gaps between the prescribers and the nurses also persist [36]. A systematic review found that AMS in nursing homes reduced antibiotic prescriptions and improved adherence to guidelines, but had no significant effect on mortality, infection rates, or hospitalization rates [37]. Integrating diagnostic stewardship into nursing education and practice is essential to strengthen knowledge of AMR, specimen handling, culture use, and interpretation of diagnostic reports [10].

This study showed that 65.8% and 39.3% of nurses either never or rarely, educate patients on the use and resistance to antibiotics. In the Arab population, limited knowledge, misconceptions, and self-medication are common, highlighting the need for public health campaigns, patient education, and stricter regulations on antibiotic distribution [38].

This study’s strengths include being the first in Oman to assess nurses’ knowledge, attitudes, and behaviours on antibiotic use and resistance, using validated ECDC instruments, a large sample, and bilingual questionnaires to enhance accessibility. However, its cross-sectional design limits causal inference, self-reported responses may introduce bias, and findings may not fully represent nurses outside the Ministry of Health, highlighting the need for further research.

## Conclusion

The results of this study highlight the need to enhance nurses’ awareness and capabilities regarding antibiotic use and antibiotic resistance. Antimicrobial resistance is a complex global issue requiring coordinated action. Nurses must be empowered through education, policy engagement, and leadership development [39,40]. Our findings show that nurses expressed a need for more education on AMR. Targeted continuing education programs may therefore enhance their competence; future research could explore the potential role of digital tools in this context. At the same time, interdisciplinary collaboration and organizational support are essential for effective behavioural change in AMS [41].

These results underscore the need for targeted education, supportive policies, and increased inclusion of nurses in AMS. Strengthening their capabilities, opportunities, and motivation is essential to empower them as frontline contributors in the global fight against antibiotic resistance.

## Supporting information

S1 Fig Topics that nurses would like to learn more about. (TIF)

S1 File Dataset. (XLSX)

S2 File Questionnaire. (PDF)

S3 File Supporting Information (S1- S5 Tables). (DOCX)

## References

[1] O’Neill J. Antimicrobial resistance: Tackling a crisis for the health and wealth of nations. Rev Antimicrob Resist. 2014.

[2] O’Neill J. Tackling drug-resistant infections globally: Final report and recommendations. 2016.

[3] GBD 2021 Antimicrobial Resistance Collaborators. Global burden of bacterial antimicrobial resistance 1990-2021: A systematic analysis with forecasts to 2050. Lancet. 2024;404(10459):1199–226. doi: 10.1016/S0140-6736(24)01867-1 39299261 PMC11718157

[4] Lewnard JA, Charani E, Gleason A, Hsu LY, Khan WA, Karkey A, et al. Burden of bacterial antimicrobial resistance in low-income and middle-income countries avertible by existing interventions: An evidence review and modelling analysis. Lancet. 2024;403(10442):2439–54. doi: 10.1016/S0140-6736(24)00862-6 38797180

[5] Pollack LA, Srinivasan A. Core elements of hospital antibiotic stewardship programs from the Centers for Disease Control and Prevention. Clin Infect Dis. 2014;59 Suppl 3(Suppl 3):S97-100. doi: 10.1093/cid/ciu542 25261548 PMC6521960

[6] Ha DR, Haste NM, Gluckstein DP. The role of antibiotic stewardship in promoting appropriate antibiotic use. Am J Lifestyle Med. 2017;13(4):376–83. doi: 10.1177/1559827617700824 31285722 PMC6600622

[7] Dellit TH, Owens RC, McGowan JE, Gerding DN, Weinstein RA, Burke JP, et al. Infectious diseases society of america and the society for healthcare epidemiology of America guidelines for developing an institutional program to enhance antimicrobial stewardship. Clin Infect Dis. 2007;44(2):159–77. doi: 10.1086/510393 17173212

[8] Olans RN, Olans RD, DeMaria A. The critical role of the staff nurse in antimicrobial stewardship--unrecognized, but already there. Clin Infect Dis. 2016;62(1):84–9. 26265496 10.1093/cid/civ697

[9] Camerini FG, Cunha TL, Fassarella CS, de Mendonça Henrique D, Fortunato JGS. Nursing strategies in antimicrobial stewardship in the hospital environment: A qualitative systematic review. BMC Nurs. 2024;23(1):147. doi: 10.1186/s12912-024-01753-y 38429699 PMC10908145

[10] Gupta R, Sharma S, Bablani V, Manocha S, Srinivasan M. Empowering nurses for effective diagnostic stewardship: An initiative to address anti-microbial resistance. Nurse Educ Pract. 2025;82:104223. doi: 10.1016/j.nepr.2024.104223 39671750

[11] Lalithabai DS, Hababeh MO, Wani TA, Aboshaiqah AE. Knowledge, attitude and beliefs of nurses regarding antibiotic use and prevention of antibiotic resistance. SAGE Open Nurs. 2022;8:23779608221076821. doi: 10.1177/23779608221076821 35600006 PMC9118425

[12] Padigos J, Ritchie S, Lim AG. Enhancing nurses’ future role in antimicrobial stewardship. Collegian. 2020;27(5):487–98. doi: 10.1016/j.colegn.2020.01.005

[13] Alriyami M, Al Omari O, Al-Daken L, Alriyami T, Al Rashdi R, Al Shukaili S, et al. Assessing knowledge of nosocomial infection among Omani student nurses: A cross-sectional study. Br J Nurs. 2022;31(2):66–70. doi: 10.12968/bjon.2022.31.2.66 35094542

[14] Merrill K, Hanson SF, Sumner S, Vento T, Veillette J, Webb B. Antimicrobial stewardship: Staff nurse knowledge and attitudes. Am J Infect Control. 2019;47(10):1219–24. doi: 10.1016/j.ajic.2019.03.022 31128981

[15] Davey K, Aveyard H. Nurses’ perceptions of their role in antimicrobial stewardship within the hospital environment. An integrative literature review. J Clin Nurs. 2022;31(21–22):3011–20. doi: 10.1111/jocn.16204 35092116 PMC9787640

[16] European Centre for Disease Prevention and Control. Survey of healthcare workers’ knowledge, attitudes and behaviours on antibiotics, antibiotic use and antibiotic resistance in the EU/EEA Stockholm: ECDC; 2019 https://www.ecdc.europa.eu/sites/default/files/documents/survey-of-healthcare-workers-knowledge-attitudes-behaviours-on-antibiotics.pdf

[17] Ashiru-Oredope D, Hopkins S, Vasandani S, Umoh E, Oloyede O, Nilsson A, et al. Healthcare workers’ knowledge, attitudes and behaviours with respect to antibiotics, antibiotic use and antibiotic resistance across 30 EU/EEA countries in 2019. Euro Surveill. 2021;26(12):1900633. doi: 10.2807/1560-7917.ES.2021.26.12.1900633 33769250 PMC7995558

[18] Al Sulayyim H, Ismail R, Hamid AA, Ghafar NA. Knowledge, attitude and practice of healthcare workers towards antibiotic resistance during the COVID-19 pandemic. JAC Antimicrob Resist. 2023;5(3):dlad068. doi: 10.1093/jacamr/dlad068 37288079 PMC10243906

[19] Sahu RK, Sahu Y. A study to assess knowledge, attitude and practices regarding antibiotic administration and its resistance among the nursing professionals working in various institute of Chhattisgarh state. Int J Sci Healthc Res. 2021;6(2):17–21.

[20] Zainaghi I, Cilluffo S, Lusignani M. Knowledge, attitudes, and practices related to antibiotic resistance among physicians and nurses in Italian intensive care: A multicenter cross-sectional survey. J Glob Antimicrob Resist. 2024;36:460–5. doi: 10.1016/j.jgar.2023.10.022 37972924

[21] Oman SO. Antimicrobial resistance (AMR) national action plan. Ministry of Health. Ministry of Agriculture & Fisheries. 2016.

[22] Manning ML, Pfeiffer J, Larson EL. Combating antibiotic resistance: The role of nursing in antibiotic stewardship. Am J Infect Control. 2016;44(12):1454–7. doi: 10.1016/j.ajic.2016.06.023 27592161

[23] Anwar M, Raziq A, Shoaib M, Baloch NS, Raza S, Sajjad B, et al. Exploring Nurses’ perception of antibiotic use and resistance: A qualitative inquiry. J Multidiscip Healthc. 2021;14:1599–608. doi: 10.2147/JMDH.S309020 34234448 PMC8254422

[24] Monsees E, Popejoy L, Jackson MA, Lee B, Goldman J. Integrating staff nurses in antibiotic stewardship: Opportunities and barriers. Am J Infect Control. 2018;46(7):737–42. doi: 10.1016/j.ajic.2018.03.028 29729830

[25] Carter EJ, Greendyke WG, Furuya EY, Srinivasan A, Shelley AN, Bothra A, et al. Exploring the nurses’ role in antibiotic stewardship: A multisite qualitative study of nurses and infection preventionists. Am J Infect Control. 2018;46(5):492–7. doi: 10.1016/j.ajic.2017.12.016 29395509 PMC6495548

[26] Sumner S, Forsyth S, Collette-Merrill K, Taylor C, Vento T, Veillette J, et al. Antibiotic stewardship: The role of clinical nurses and nurse educators. Nurse Educ Today. 2018;60:157–60. doi: 10.1016/j.nedt.2017.10.011 29132017

[27] Chater A, Courtenay M. Community nursing and antibiotic stewardship: The importance of communication and training. Br J Community Nurs. 2019;24(7):338–42. doi: 10.12968/bjcn.2019.24.7.338 31265341

[28] Fisher CC, Cox VC, Gorman SK, Lesko N, Holdsworth K, Delaney N, et al. A theory-informed assessment of the barriers and facilitators to nurse-driven antimicrobial stewardship. Am J Infect Control. 2018;46(12):1365–9. doi: 10.1016/j.ajic.2018.05.020 30077436

[29] Carter EJ, Manning ML, Pogorzelska-Maziarz M. Clinical Nurse Preparation and Partnership in Antibiotic Stewardship Programs: National survey findings are a call to action for nurse leaders. J Nurs Adm. 2019;49(12):591–5. doi: 10.1097/NNA.0000000000000821 31725058

[30] Organization WH. WHO competency framework for health workers’ education and training on antimicrobial resistance. WHO competency framework for health workers’ education and training on antimicrobial resistance 2018.

[31] CfDCaP C. Antibiotic Stewardship Trainings. https://www.cdc.gov/antibiotic-use/hcp/training/index.html. 2024.

[32] Chater AM, Family H, Abraao LM, Burnett E, Castro-Sanchez E, Du Toit B, et al. Influences on nurses’ engagement in antimicrobial stewardship behaviours: A multi-country survey using the Theoretical Domains Framework. J Hosp Infect. 2022;129:171–80. doi: 10.1016/j.jhin.2022.07.010 35843415

[33] van Huizen P, Kuhn L, Russo PL, Connell CJ. The nurses’ role in antimicrobial stewardship: A scoping review. Int J Nurs Stud. 2021;113:103772. doi: 10.1016/j.ijnurstu.2020.103772 33080476

[34] Kirby E, Broom A, Overton K, Kenny K, Post JJ, Broom J. Reconsidering the nursing role in antimicrobial stewardship: A multisite qualitative interview study. BMJ Open. 2020;10(10):e042321. doi: 10.1136/bmjopen-2020-042321 33122328 PMC7597488

[35] Chandramohan S, Al-Mohaithef M, Hazazi A, Elsayed EH. Knowledge and perceptions on hand hygiene among nurses in the Asir region, Kingdom of Saudi Arabia. Saudi J Health Sci. 2020;9(1):30. doi: 10.4103/sjhs.sjhs_58_19

[36] McElligott M, Welham G, Pop-Vicas A, et al. Antibiotic stewardship in nursing facilities. Infectious Disease Clinics. 2017;31(4):619–38. 29079152 10.1016/j.idc.2017.07.008

[37] Feldstein D, Sloane PD, Feltner C. Antibiotic stewardship programs in nursing homes: A systematic review. J Am Med Dir Assoc. 2018;19(2):110–6. doi: 10.1016/j.jamda.2017.06.019 28797590

[38] Hassan BAR, Mohammed AH, AL-Jewari WM, Blebil A, Dujaili J, Wayyes AM, et al. Knowledge and attitude towards antibiotic use and resistance among Arab population: A questionnaire-based study of 11 countries from the Middle East and North Africa. Journal of Pharmaceutical Health Services Research. 2023;14(2):131–40. doi: 10.1093/jphsr/rmad014

[39] Almanfaluthi M. Empowering nurses as key players in the regional fight against antimicrobial resistance. PSHMS. 2025;6:183–90. doi: 10.30595/pshms.v6i.1422

[40] Wiley KC, Villamizar HJ. Antibiotic resistance policy and the stewardship role of the nurse. Policy Polit Nurs Pract. 2019;20(1):8–17. doi: 10.1177/1527154418819251 30541388

[41] Danielis M, Buttiron Webber T, Barchielli C, Mongardi M, Regano D. Unveiling antimicrobial stewardship competence among Italian nurses: results from a nationwide survey. Antimicrob Resist Infect Control. 2025;14(1):16. doi: 10.1186/s13756-025-01531-8 39988671 PMC11849391

