# Supplementary material for: Knowledge, attitudes and behaviours of nurses about antibiotic use and antibiotic resistance in Oman
Source: PLoS One. 2026 May 18;21(5):e0342371. doi: 10.1371/journal.pone.0342371 (PMC13183194; doi:10.1371/journal.pone.0342371)
Supplement: S2 File — (PDF) [file pone.0342371.s003.pdf]

Survey of Nurses' Knowledge and attitudes about Antibiotics and antibiotic resistance and Sources of information in Oman

➢ **Demographic Section**

1. **Please specify in which governorate you practice**
    - Muscat
    - Dhofar
    - Musandam
    - Al Buraymi
    - Ash Sharqiyah South
    - Ash Sharqiyah North
    - Al Batinah South
    - Al Batinah North
    - Ad Dakhiliyah
    - Ad Dhahirah
    - Al Wusta
2. **What is your predominant role? (i.e.>50% of your time)?**
    - General Nurse
    - BSc. Nurse
    - BSc. Nurse with Specialist
3. **Where do you predominantly practice? (i.e. >50% of your time):**
    - Hospital (any hospital type)
    - Secondary care center (polyclinic or dialysis center)
    - Primary health center.
4. **How many years have you been practicing in your current profession?**
    - 0-2 years
    - 3-5 years
    - 6-10 years
    - 11-15 years
    - 16-20 years
    - 21-25 years
    - >25 years
5. **What is your age?**
    - 22-25 years
    - 26-35 years
    - 36-45 years
    - 46-55 years
    - 56-65 years
    - >66 years

6. **What gender do you identify with?**
    - Male
    - Female

➢ ***Knowledge about antibiotic use and antibiotic resistance***

7. **Please answer whether you believe these statements are true or false. If you are not sure, then please add this response**

	**correct answer**	**True**	**False**	**Unsure**
Antibiotics are effective against viruses	False			
Antibiotics are effective against cold infections	False			
Unnecessary use of antibiotics makes them become ineffective	True			
Taking antibiotics has associated side effects or risks such as diarrhea, colitis, allergies	True			
Every person treated with antibiotics is at an increased risk of antibiotic resistant infection	True			
Antibiotic resistant bacteria can spread from person to person	True			
Healthy people can carry antibiotic resistant bacteria	True			
The use of antibiotics to stimulate growth in farm animals is legal in Oman	False			

❖ ***For the next questions, to what extent do you agree or disagree with the following statements:***

	**Strongly disagree**	**Disagree**	**Undecided**	**Agree**	**Strongly agree**	**I do not understand the question**	**Not Applicable**
**Perceived Knowledge**							

I know what antibiotic resistance is							
I know what information to give to individuals about prudent use of antibiotics and antibiotic resistance							
I have sufficient knowledge about how to use antibiotics appropriately for my current practice							
**Opportunity**							
I have easy access to guidelines I need on managing infections							
I have easy access to the materials I need to give advice on prudent antibiotic use and antibiotic resistance							
I have good opportunities to provide advice on prudent antibiotic use to individuals							
**Motivation to initiate antibiotic prescriptions**							
I know there is a connection between my administering of antibiotics and emergence and spread of antibiotic-resistant bacteria							
I have a key role in helping control antibiotic resistance							
**One Health: environmental and animal health factors that are important in contributing to antibiotic resistance in bacteria from humans**							
Environmental factors such as wastewater in the environment are important in contributing to antibiotic resistance in bacteria from humans							
Excessive use of antibiotics in livestock and food production is important in contributing to antibiotic resistance in bacteria from humans							

❖ ***Please answer the following questions, considering the last one week only in your clinical practice:***

8. How often did you administer antibiotics during the last one week?

- Once a day
- More than once a day
- Once a week
- More than once a week

- rarely
- never
- I do not remember
- Not Applicable

9. **How often did you give out resources (e.g. leaflets or pamphlets) on prudent antibiotic use or management of infections to individuals during the last one week?**

- Once a day
- More than once a day
- Once a week
- More than once a week
- rarely
- never
- I do not remember
- Not Applicable

10. **How often did you give out advice related to prudent antibiotic use or management of infections to an individual during the last one week?**

- Once a day
- More than once a day
- Once a week
- More than once a week
- Rarely
- Never
- I do not remember
- Not Applicable

11. **If you were not able to give out advice or resources as frequently as you administer antibiotics, why was this?**

- Patient does not require information
- Difficulty getting patient to understand diagnosis
- Patient uninterested in information
- No resources available
- Insufficient time
- I was able to give out advice or resources as needed
- Language barriers
- I was not sure what advice to provide
- Not applicable

➢ ***Sources of information about avoiding unnecessary dispensing of antibiotics Section***

12. **Which of the following social media networks do you mainly use for professional activities?**

(Choose all that apply)

- Twitter
- Facebook
- LinkedIn
- Google+
- YouTube
- Instagram
- I do not use social media
- Others____

13. In the last 12 months, do you remember receiving any information about avoiding unnecessary administration of antibiotics?

- Yes
- No
- Unsure

14. Did the information contribute to changing your views about avoiding unnecessary administration of antibiotics?

- Yes
- No
- Unsure

15. On the basis of the information you received, have you changed your practice on the administration of antibiotics?

- Yes
- No
- Unsure

16. How did you first get this information about avoiding unnecessary administration of antibiotics? (Select all that apply).

- Colleague or peer
- My workplace
- Media (TV/Radio) adverts
- Social Media
- Newspaper
- Published guidelines
- Training - conference/group
- Training - one to one
- Government policy
- Scientific organization
- My medical professional body
- Audit and feedback

- Others: ____

17. Which source(s) of information has had the most influence on changing your views? Select no more than 2.

- Colleague or peer
- My workplace
- Media (TV/radio) adverts
- Social media
- Newspaper
- Published guidelines
- Training - conference/group
- Training - one to one
- Government policy
- Scientific organization
- My medical professional body
- Audit and feedback
- Others: ____

➢ ***Awareness of initiatives and national action plans on antimicrobial resistance Section***

18. What initiatives are you aware of which focus on antibiotic awareness and resistance? Select all that apply

- TV or Radio advertising for the public
- Toolkits and resources for healthcare workers
- National or regional guidelines on management of infections
- Awareness raising from professional organizations
- Conference/Events focused on tackling antibiotic resistance
- National or regional posters or leaflets on antibiotic awareness
- Newspaper (national) articles on antibiotic resistance
- World Antibiotic Awareness Week
- I am not aware of any initiatives

19. Does your country have a national action plan on antimicrobial resistance?

- Yes
- No
- Unsure

20. In the management of infections, which of these do you use regularly? (Choose all that apply)

- Clinical practice guidelines
- Documentation from the pharmaceutical industry
- Medical representatives from industry
- Previous clinical experience

- Continuing education training courses
- Infection specialists
- Scientific journals
- Professional resources/publications
- Social media
- None of the above
- I do not know
- Others: ____

21. On which topics would you like to receive more information? (Choose all that apply)

- Resistance to antibiotics
- How to use antibiotics
- Medical conditions for which antibiotics are used
- Prescription of antibiotics
- Links between the health of humans, animals and the environment
- None
- Others: ______________________________________
