# Supplementary material for: Knowledge, attitudes and behaviours of nurses about antibiotic use and antibiotic resistance in Oman
Source: PLoS One. 2026 May 18;21(5):e0342371. doi: 10.1371/journal.pone.0342371 (PMC13183194; doi:10.1371/journal.pone.0342371)
Supplement: S3 File — (DOCX) [file pone.0342371.s004.docx]

**S1 Table. Univariable linear regression of the relationship between knowledge scores and demographic characteristics of respondents.**

|  |  | Unstandardized Coefficients | | Standardized Coefficients | t | Sig. | 95.0% Confidence Interval for B | |
| --- | --- | --- | --- | --- | --- | --- | --- | --- |
|  |  | **B** | **Std. Error** | **Beta** |  |  | **Lower Bound** | **Upper Bound** |
| Gender | Female |  |  |  |  |  |  |  |
|  | Male | 0.590 | 0.211 | 0.135 | 2.794 | **0.005** | 0.175 | 1.005 |
| Age | 24-35 years |  |  |  |  |  |  |  |
|  | 36-45 years | 0.578 | 0.145 | 0.195 | 3.981 | **< 0.001** | 0.293 | 0.863 |
|  | 46-55 years | 0.985 | 0.310 | 0.155 | 3.174 | **0.002** | 0.375 | 1.595 |
|  | 56-65 years | -0.290 | 0.652 | -0.021 | -0.445 | 0.657 | -1.571 | 0.991 |
| Governorate | Ad Dakhiliyah |  |  |  |  |  |  |  |
|  | Ad Dhahirah | 1.000 | 0.532 | 0.098 | 1.881 | 0.061 | -0.045 | 2.045 |
|  | Al Batinah North | 0.500 | 0.471 | 0.056 | 1.061 | 0.289 | -0.426 | 1.426 |
|  | Al Batinah South | 0.286 | 0.379 | 0.042 | 0.753 | 0.452 | -0.460 | 1.031 |
|  | Al Buraymi | 0.062 | 0.420 | 0.008 | 0.149 | 0.882 | -0.763 | 0.888 |
|  | Al Wusta | 0.424 | 0.277 | 0.100 | 1.529 | 0.127 | -0.121 | 0.968 |
|  | Ash Sharqiyah North | 0.000 | 0.636 | 0.000 | 0.000 | 1.000 | -1.250 | 1.250 |
|  | Ash Sharqiyah South | 0.156 | 0.329 | 0.028 | 0.475 | 0.635 | -0.490 | 0.802 |
|  | Dhofar | 0.500 | 0.766 | 0.033 | 0.653 | 0.514 | -1.005 | 2.005 |
|  | Musandam | 0.167 | 0.471 | 0.019 | 0.354 | 0.724 | -0.759 | 1.093 |
|  | Muscat | 0.136 | 0.225 | 0.046 | 0.605 | 0.545 | -0.307 | 0.579 |
| Profession | BSc. Nurse |  |  |  |  |  |  |  |
|  | BSc. Nurse with Specialism | 0.574 | 0.249 | 0.128 | 2.305 | **0.022** | 0.084 | 1.063 |
|  | General Nurse (Diploma) | -0.080 | 0.170 | -0.026 | -0.472 | 0.637 | -0.414 | 0.253 |
| Place of practice | Hospital (any hospital type) |  |  |  |  |  |  |  |
|  | Primary health center | 0.060 | 0.198 | 0.015 | 0.302 | 0.763 | -0.329 | 0.449 |
|  | Secondary care center (polyclinic or dialysis center) | -0.341 | 0.402 | -0.041 | -0.847 | 0.397 | -1.131 | 0.449 |
| Years of practice | 0-2 years |  |  |  |  |  |  |  |
|  | 3-5 years | 0.581 | 0.350 | 0.124 | 1.658 | 0.098 | -0.108 | 1.270 |
|  | 6-10 years | 0.519 | 0.314 | 0.152 | 1.652 | 0.099 | -0.099 | 1.137 |
|  | 11-15 years | 0.620 | 0.307 | 0.196 | 2.019 | **0.044** | 0.016 | 1.224 |
|  | 16-20 years | 1.303 | 0.321 | 0.355 | 4.056 | **< 0.001** | 0.672 | 1.935 |
|  | 21-25 years | 1.205 | 0.421 | 0.178 | 2.865 | **0.004** | 0.378 | 2.032 |
|  | >25 years | 1.705 | 0.649 | 0.137 | 2.626 | **0.009** | 0.429 | 2.982 |
| *B* = unstandardized coefficient; *SE* = standard error; beta = standardized coefficient; t = t-statistic; p = p-value: CI = confidence interval. | | | | | | | | |

**S2 Table. Association between demographic characteristics and knowledge scores (0–8) among nursing staff (n=424).**

| Variable |  |  | Score (Range 0–8) | No. of Respondents (%) | p-value |
| --- | --- | --- | --- | --- | --- |
| **Gender** | Female | Median (IQR) | 5.0 (4.0 to 6.0) | 369 (87.0) |  |
|  | Male | Median (IQR) | 6.0 (5.0 to 7.0) | 55 (13.0) | ***0.008*** |
| **Age** | 24-35 years | Median (IQR) | 5.0 (4.0 to 6.0) | 209 (49.3) |  |
|  | 36-45 years | Median (IQR) | 5.0 (5.0 to 6.0) | 186 (43.9) |  |
|  | 46-55 years | Median (IQR) | 6.0 (5.0 to 7.0) | 24 (5.7) | ***<0.001*** |
|  | 56-65 years | Median (IQR) | 4.0 (4.0 to 5.0) | 5 (1.2) |  |
| **Governorate** | Ad Dakhiliyah | Median (IQR) | 5.0 (3.0 to 6.0) | 55 (13.0) | 0.931 |
|  | Ad Dhahirah | Median (IQR) | 5.0 (5.0 to 7.0) | 9 (2.1) |  |
|  | Al Batinah North | Median (IQR) | 5.0 (4.0 to 7.0) | 12 (2.8) |  |
|  | Al Batinah South | Median (IQR) | 5.0 (4.0 to 6.0) | 21 (5.0) |  |
|  | Al Buraymi | Median (IQR) | 5.0 (4.0 to 6.0) | 16 (3.8) |  |
|  | Al Wusta | Median (IQR) | 5.0 (5.0 to 6.0) | 59 (13.9) |  |
|  | Ash Sharqiyah North | Median (IQR) | 5.5 (4.2 to 6.0) | 6 (1.4) |  |
|  | Ash Sharqiyah South | Median (IQR) | 5.0 (4.0 to 6.0) | 32 (7.5) |  |
|  | Dhofar | Median (IQR) | 5.5 (4.0 to 7.0) | 4 (0.9) |  |
|  | Musandam | Median (IQR) | 5.0 (4.0 to 7.0) | 12 (2.8) |  |
|  | Muscat | Median (IQR) | 5.0 (4.0 to 6.0) | 198 (46.7) |  |
| **Profession** | Bs. Nurse | Median (IQR) | 5.0 (4.0 to 6.0) | 102 (24.1) |  |
|  | Bs. Nurse with Specialist | Median (IQR) | 6.0 (5.0 to 7.0) | 52 (12.3) | ***0.008*** |
|  | General Nurse | Median (IQR) | 5.0 (4.0 to 6.0) | 270 (63.7) |  |
| **Place of practice** | Hospital (any hospital type) | Median (IQR) | 5.0 (4.0 to 6.0) | 344 (81.1) | 0.446 |
|  | Primary health center | Median (IQR) | 5.0 (5.0 to 6.0) | 66 (15.6) |  |
|  | Secondary care center (polyclinic or dialysis center) | Median (IQR) | 5.0 (4.0 to 5.0) | 14 (3.3) |  |
| **Year of practice** | 0-2 years | Median (IQR) | 5.0 (3.2 to 5.0) | 26 (6.1) |  |
|  | 3-5 years | Median (IQR) | 5.0 (4.0 to 6.0) | 47 (11.1) |  |
|  | 6-10 years | Median (IQR) | 5.0 (4.0 to 6.0) | 104 (24.5) |  |
|  | 11-15 years | Median (IQR) | 5.0 (4.0 to 6.0) | 135 (31.8) |  |
|  | 16-20 years | Median (IQR) | 6.0 (5.0 to 7.0) | 85 (20.0) | ***< 0.001*** |
|  | 21-25 years | Median (IQR) | 5.0 (5.0 to 7.0) | 21 (5.0) |  |
|  | >25 years | Median (IQR) | 6.5 (5.2 to 7.0) | 6 (1.4) |  |
| **Mann-Whitney U test, Kruskal-Wallis 1-way ANOVA test** | | | | | |

**S3 Table. Comparison of demographic factors between participants with full and incomplete knowledge.**

| Variable | No^1^, n = 409 (%) | Yes^1^, n = 15 (%) | p-value^2^ |
| --- | --- | --- | --- |
| **Gender** |  |  |  |
| Female | 360 (88.0) | 9 (60.0) |  |
| Male | 49 (12.0) | 6 (40.0) | ***0.007*** |
| **Age** |  |  |  |
| 24-35 years | 206 (50.4) | 3 (20.0) |  |
| 36-45 years | 177 (43.3) | 9 (60.0) |  |
| 46-55 years | 21 (5.1) | 3 (20.0) | **0.024** |
| 56-65 years | 5 (1.2) | 0 (0.0) |  |
| **Governorate** |  |  | **0.036** |
| Ad Dakhiliyah | 51 (12.5) | 4 (26.7) |  |
| Ad Dhahirah | 7 (1.7) | 2 (13.3) |  |
| Al Batinah North | 12 (2.9) | 0 (0.0) |  |
| Al Batinah South | 19 (4.6) | 2 (13.3) |  |
| Al Buraymi | 16 (3.9) | 0 (0.0) |  |
| Al Wusta | 59 (14.4) | 0 (0.0) |  |
| Ash Sharqiyah North | 6 (1.5) | 0 (0.0) |  |
| Ash Sharqiyah South | 32 (7.8) | 0 (0.0) |  |
| Dhofar | 4 (1.1) | 0 (0.0) |  |
| Musandam | 11 (2.7) | 1 (6.7) |  |
| Muscat | 192 (46.9) | 6 (40.0) |  |
| **Profession** |  |  | 0.223 |
| Bs. Nurse | 99 (24.2) | 3 (20.0) |  |
| Bs. Nurse with Specialist | 48 (11.7) | 4 (26.7) |  |
| General Nurse | 262 (64.1) | 8 (53.3) |  |
| **Place of practice** |  |  | 0.733 |
| Hospital (any hospital type) | 331 (81.0) | 13 (86.7) |  |
| Primary health center | 64 (15.6) | 2 (13.3) |  |
| Secondary care center (polyclinic or dialysis center) | 14 (3.4) | 0 (0.0) |  |
| **Years of practice** |  |  | 0.168 |
| 0-2 years | 26 (6.4) | 0 (0.0) |  |
| 3-5 years | 45 (11.0) | 2 (13.3) |  |
| 6-10 years | 102 (24.9) | 2 (13.3) |  |
| 11-15 years | 132 (32.3) | 3 (20.0) |  |
| 16-20 years | 80 (19.6) | 5 (33.3) |  |
| 21-25 years | 19 (4.6) | 2 (13.3) |  |
| >25 years | 5 (1.2) | 1 (6.7) |  |
| **^1^ Yes: Full knowledge and answered all questions correctly, No: participants got less than full score;^2^Fisher's exact test, Chi-Square test** | | | |

**S4 Table. Awareness of initiatives on antibiotic awareness and resistance.**

| Items | n (%) |
| --- | --- |
| TV or radio advertising for the public | 96 (22.6) |
| Toolkits and resources for healthcare workers | 137 (32.3) |
| National or regional guidelines on management of infections | 164 (38.7) |
| Awareness raising from professional organizations | 184 (43.4) |
| Conference/Events focused on tackling antibiotic resistance | 118 (27.8) |
| National or regional posters or leaflets on antibiotic awareness | 109 (25.7) |
| Newspaper (national) articles on antibiotic resistance | 38 (9.0) |
| World Antibiotic Awareness Week | 98 (23.1) |
| I am not aware of any initiatives | 45 (10.6) |

| In the management of infections, which of these do you use regularly? | n (%) |
| --- | --- |
| Clinical practice guidelines | 249 (58.7) |
| Documentation from the pharmaceutical industry | 87 (20.5) |
| Medical representatives from industry | 27 (6.4) |
| Previous clinical experience | 146 (34.4) |
| Continuing education training courses | 155 (36.6) |
| Infection specialists | 148 (34.9) |
| Scientific journals | 36 (8.5) |
| Professional resources/publications | 84 (19.8) |
| Social media | 96 (22.6) |
| None of the above | 12 (2.8) |
| I Do Not Know | 18 (4.2) |
| Others | 14 (3.3) |

**S5 Table. Resources used for the management of patients with infections.**

**S1 Fig**. **Topics that nurses would like to learn more about.**
